# Primary extraskeletal osteosarcoma of the post-hepatic caudal vena cava in a dog—Case report

**DOI:** 10.3389/fvets.2023.1197236

**Published:** 2023-09-21

**Authors:** Giovanni Tremolada, Lynn Griffin, Alison C. Manchester, Tawfik Aboellail, Janis M. Lapsley, Laura E. Selmic

**Affiliations:** ^1^Flint Animal Cancer Center, Colorado State University College of Veterinary Medicine, Fort Collins, CO, United States; ^2^Department of Clinical Sciences, Colorado State University College of Veterinary Medicine, Fort Collins, CO, United States; ^3^Department of Veterinary Clinical Sciences, The Ohio State University, Columbus, OH, United States

**Keywords:** osteosarcoma, extraskeletal osteosarcoma, vena cava, canine, dog

## Abstract

Extraskeletal osteosarcoma (EOSA) in dogs is a rare malignant mesenchymal tumor of somatic soft tissues or more commonly visceral organs with a poor prognosis. In dogs, EOSAs have been described as arising from multiple locations, but differently from humans, never from a main vessel. In this report, we describe the first case of an EOSA arising from the post-hepatic caudal vena cava in a 7-year-old male neutered mix breed dog. This report focuses on the description of the diagnostic challenges to obtain a preoperative diagnosis, highlights the importance of histopathology for a correct diagnosis, and introduces a new differential diagnosis for an animal presenting with a suspected thrombus of the vena cava.

## Introduction

Extraskeletal osteosarcoma (EOSA) in dogs is a rare malignant mesenchymal tumor of somatic soft tissues or more commonly visceral organs that is characterized by the production of osteoid without involvement of bone or periosteal tissue. An exception to this definition is mixed malignant tumors of the mammary gland and thyroid (carcinosarcomas), where osteoid production is considered to be related to stromal metaplasia associated with adenocarcinomas (1). EOSA is an aggressive tumor with a reported median survival time ranging from 26 to 190 days, depending on the treatment received (2–4). In dogs, EOSAs have been described arising from multiple locations, such as the small intestine (1–3), spleen (1, 4), liver (3), mesenteric root (4, 5), eye (6), heart (7), meninges (8), salivary gland (9), lung (10), adrenal gland (1), testicle (1), and vagina (1), or developed secondary to chronic inflammation from gossypiboma (11, 12).

In humans, EOSA is also a rare tumor and tends to arise more commonly from the soft tissue of the extremities rather than from visceral organs (1). A few published reports in humans do describe primary EOSAs originating from the pulmonary artery (13, 14), whereas, to the best of the authors' knowledge, there are no published reports describing EOSA arising from major blood vessels in veterinary patients.

The purpose of this case report was to describe an EOSA arising from the post-hepatic caudal vena cava in a dog, adding to the list of the differential diagnoses that ought to be considered and highlighting the need to biopsy the mass for obtaining a correct diagnosis.

## Case presentation

A 7-year-old, 6.4-kg male neutered mixed-breed dog was presented to his primary veterinarian for a 5-day history of anorexia, polyuria/polydipsia, non-specific gastrointestinal signs, and a rapid onset of abdominal distension. Physical examination was unremarkable, except for moderate to marked abdominal distension and the presence of palpable fluid wave. A serum biochemistry analysis, complete blood count (CBC), canine-specific pancreatic lipase, pre-prandial bile acids, and urinalysis were performed, and findings are reported in Table 1. Urinalysis showed isosthenuria (1.016) with a trace of protein and a negative sediment.

**Table 1:** Timeline of blood work changes noted before surgical intervention was attempted to remove the suspected caval thrombus.

	**CBC**	**Chemistry/ blood gas analysis**	**Canine specific pancreatic lipase**	**Bile acids**	**Texas GI panel**	**Basal cortisol**	**Coagulation panel**
Visit 1	Normal	Albumin 2.4 g/dl (range 2.7–3.9 g/dl) Globulin 1.9 g/dl (range 2.4–4 g/dl) ALT 589 IU/L (range 18–121 IU) AST 91 IU/L (range 16–55 IU/L) Cholesterol 112 mg/dl (range 131–345 mg/dl) Sodium 140 mmol/L (range 142–152 mmol/L)	234 μg/L, normal range 0–200 μg/L	Pre-prandial: 63.5 μmol/ (normal value <7 μmol/L)	–	–	–
Visit 2	Reticulocytes 139 × 10^3^/μl (range 10–110 × 10^3^/μl)	Albumin 2 g/dl (range 2.7–3.9 g/dl) Globulin 1.8 g/dl (range 2.4–4 g/dl) ALT 202 IU/L (range 18–121 IU) Cholesterol 110 mg/dl (range 131–345 mg/dl) Glucose 140 mg/dl (range 63–114 mg/dl) Calcium 8.2 mg/dl (range 8.4–11.8 mg/dl) Triglycerides 208 mg/dl (range 20–150 mg/dl) BUN 7 mg/dl (range 9–31 mg/dl)	Reported normal—no value available from records	168.3 mmol/l	–	–	–
Visit 3	Hematocrit 37% (range 40–55%); reticulocytes 122.5 × 10^3^/μl, (range 0–110 × 10^3^/μl), neutrophils 11.1 × 10^3^/μl, (range 2.6–11 × 10^3^/μl)	Total protein 4.9 g/dl (range 5–7 g/dl) Sodium 2.8mEq/L (range 4.1–5.6 mEq/L)	–	Pre-prandial: 111 μmol/L (normal value <7 μmol/L)	Folate: 6.6 μg/L, (range 7.7–24.4 μl/L)	5.3 μg/dl, (range 1.6–2 μg/dl)	–
Visit 4		Albumin 2.9 g/dl (range 3–4.3 g/dl)	–	–	–	–	Activated partial thromboplastin time APTT 15.7 s, (range 9.4–15 s), D-dimers 715 ng/ml (range 15–125 ng/ml) fibrinogen 85 mg/ml (range 100–300 mg/ml)
Visit 5	Reticulocytes 122.7 × 10^3^/μl (range 0–100 × 10^3^/μl)	Total protein 4.1 g/dl (range 5–7 g/dl) albumin 2.3 g/dl (range 3–4.3 g/dl) ALT 91 IU (range 10–90 IU)	–	–	–	–	Normal

APTT, activated partial thromboplastin time.

Due to the abnormal liver values, gastrointestinal symptoms, and the suspected presence of abdominal effusion, the dog was started on supportive therapy [denamarin (32 mg/kg twice a day (BID), maropitant (2.2 mg/kg once a day (SID), and omeprazole (unknown dose and frequency)] and was referred to a specialty hospital for an abdominal ultrasound. An abdominal ultrasound was performed by a board-certified veterinary radiologist and showed changes consistent with non-specific hepatopathy, pancreatitis, and a single small splenic nodule. The remainder of the ultrasonogram was considered normal. An abdominocentesis was performed using a 25-g needle, and 1 ml of pink, hazy fluid was collected and submitted for fluid analysis. Fluid analysis was consistent with a transudate (total protein 1.7 g/dl; nucleated cells 200/μl) with a mixed population of reactive mesothelial cells, macrophages, and degenerate neutrophils. No neoplastic cells were identified.

Based on the results of the tests, a tentative diagnosis of non-specific hepatopathy and pancreatitis was made.

The dog was returned to the primary veterinarian 2 days after the visit to the specialty hospital to have bloodwork repeated. CBC, serum biochemistry, canine-specific pancreatic lipase, and bile acid values are reported in Table 1. Bile acids were increased from the previous measurement, but it was unclear whether the animal was fasting during the test or not. The dog was started on spironolactone (0.9 mg/kg BID orally) to help reduce the volume of the abdominal effusion.

Due to a lack of improvement in clinical signs 3 weeks after the initial visit to the primary veterinarian, the dog was referred to Colorado State University (CSU) internal medicine service for a second opinion. On physical examination, the main abnormalities noted were a severely distended abdomen with prominent subcutaneous vessels of the abdominal wall, mild dental tartar, and soft stools on rectal examination. Bloodwork comprising a CBC, total protein, bile acids, blood gas analysis, Texas GI panel, and basal cortisol was performed. CBC showed mild regenerative anemia and mild neutrophilia. Total protein level was improved from the previous examination, making hypoalbuminemia unlikely to be the cause of the ascites. Pre-prandial bile acids were increased. The blood gas analysis showed marked hypokalemia, likely related to the chronic abdominal effusion. The Texas GI panel showed low folate, possibly indicative of proximal intestinal malabsorption, while basal cortisol was normal (Table 1).

The dog was discharged with the plan to continue with the diuretic therapy and to reduce the frequency of Denamarin administration.

Two weeks after the initial visit (5 weeks after the initial presentation), the dog returned to CSU to have an abdominal ultrasound, and liver biopsies were performed.

**Figure 1:**
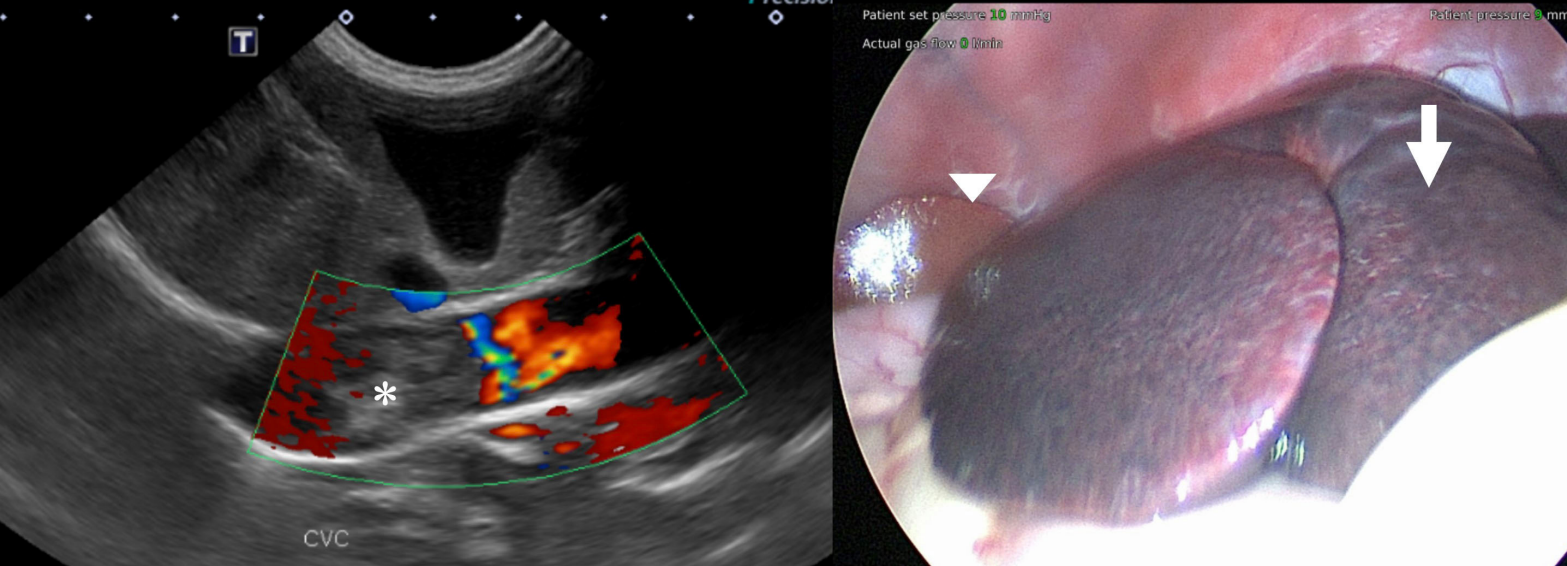
**(Left)** Color flow Doppler image of the suspected caudal vena cava thrombus showing no evidence of blood flow within it (asterisk). **(Right)** Laparoscopic view of the liver before biopsies. Note the congested liver parenchyma of the left liver lobes (arrow) compared to the normal parenchyma of the right side (arrowhead).

Blood for serum biochemistry and coagulation panel was collected before the abdominal ultrasound. The results showed persistent hypoalbuminemia and a mild elevation of activated partial thromboplastin time, elevated D-dimers, and a low quantitative fibrinogen. The dog was sedated, and 1 L of serosanguinous fluid was drained from the abdomen to facilitate imaging but not submitted for analysis. An abdominal ultrasound focused on the liver and hepatic vasculature identified diffusely hyperechoic and heterogeneous liver parenchyma and a large echogenic post-hepatic thrombus in the vena cava. The absence of blood flow was identified within the thrombosed lumen on Doppler interrogation (Figure 1, left). Cytology of the liver was performed and revealed hepatocellular hyperplasia with mononuclear inflammation and the absence of overt neoplasia. The dog was subsequentially anesthetized, and laparoscopic liver biopsies for histopathology, copper and iron quantification, and tissue culture were performed. On laparoscopic examination of the abdomen, the left division of the liver appeared congested (Figure 1, right). Biopsies of the liver, duodenum, jejunum, and ileum were performed. Histopathology of the liver revealed marked centrilobular congestion with chronic lymphoplasmacytic inflammation and periportal fibrosis, and mild-to-moderate lymphoplasmacytic/eosinophilic inflammation and mild ectasia of mucosal lymphatics were found in the intestine. The dog recovered well from the procedures and was discharged from the hospital the following day on clopidogrel (3.5 mg/kg SID) and an increased dose of spironolactone (2.3 mg/kg in the morning and 1.1 mg/kg in the evening).

Supplementation of vitamin B12 (100 mcg/kg SID) and folates (75 mg/kg SID) and a newly selected protein diet (Royal Canin potato and fish) were started to treat the suspected lymphangiectasia. Liver metal analysis revealed an increased iron value (2,250 ppm, normal range 400–1,200 ppm) and normal copper value (336 ppm, normal range 120–400 ppm). No bacterial growth was identified on the liver biopsy culture.

The dog's ascites did not improve after 2 weeks from starting the new therapy. Apixaban (0.25 mg/kg BID) was added to help resolve the caudal vena cava thrombus. Still, the clinical signs did not improve and an abdominocentesis had to be performed weekly, removing 300–500 ml of fluid each time. An abdominal ultrasound was repeated a month after initiating medical therapy and did not show any reduction in the size of the suspected thrombus. Surgical removal of the thrombus or caudal vena cava stenting was re-offered as a treatment option but declined by the owner due to financial constraints. The dog was continued on the same medical therapy previously prescribed.

**Figure 2:**
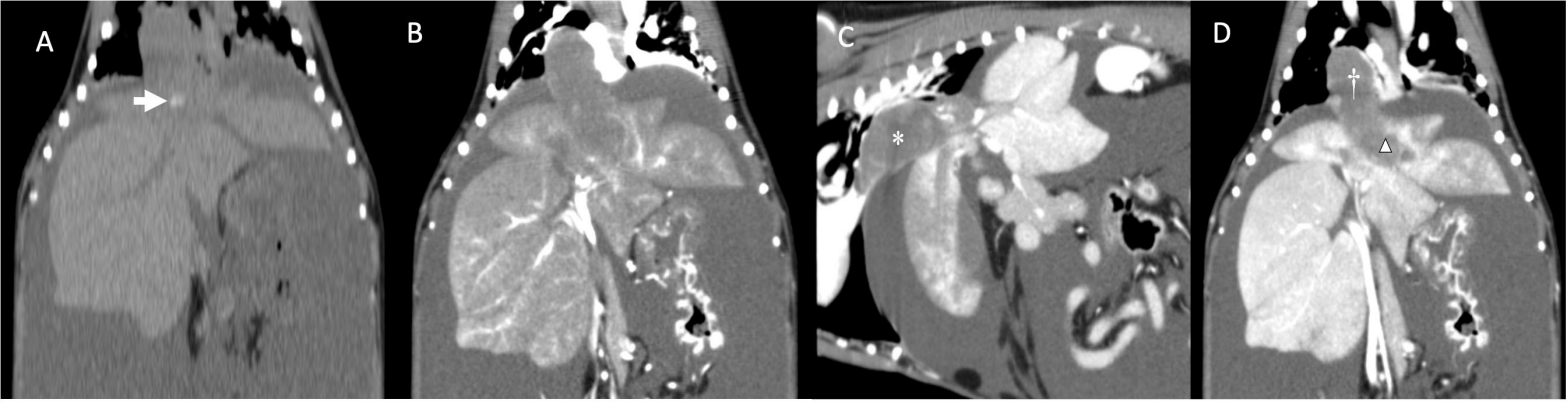
**(A)** Dorsal reconstruction of dog's computed tomography before administration of contrast medium. Note the mineralization inside the mass/thrombus (arrow). **(B)** Dorsal reconstruction of dog's computed tomography (arterial phase). Note the minimal contrast enhancement compared to **(A)**. **(C)** Sagittal reconstruction of dog's computed tomography showing a large mass/thrombus (asterisk) completely obstructing the caudal vena cava. **(D)** Dorsal reconstruction of the dog's CT showing the large mass/thrombus occupying the caudal vena cava (cross) and invading into the left hepatic vein (triangle). Note the patchy appearance of the congested left liver lobes. No parenchymal invasion of the mass is noted.

One month after the last appointment (9 weeks after the initial presentation), because of the persistent severe ascites requiring frequent abdominocentesis, the owner elected to have a computed tomography (CT) scan of the abdomen performed in preparation for surgical thrombectomy. The dog's physical examination was unchanged. CBC, serum biochemistry, and coagulation panel were repeated. The CBC showed an increase in the reticulocyte count. The serum biochemistry showed hypoproteinemia and hypoalbuminemia and a minimally increased ALT. Activated partial thromboplastin time and prothrombin time were normal. The dog was also blood-typed and was DEA 1.1-negative. Thoracic radiographs were obtained before surgery and showed a mild diffuse interstitial pattern throughout the pulmonary parenchyma, an enlarged caudal vena cava, and no evidence of metastatic disease. The dog was anesthetized, and a CT examination with an angiogram of the abdomen revealed the presence of a large amount of mixed soft tissue/mineral attenuating material in the caudal vena cava and left hepatic vein (Figure 2). The thrombus extended from the liver to the right atrium, with minimal contrast enhancement noted compared to the pre-contrast images. Patchy contrast enhancement of the left lateral, left medial, quadrate, and right medial liver lobes, a small conglomeration of vessels ventral to the azygos vein communicating with the portal circulation, caudal vena cava duplication at the level of the left renal vein, and marked abdominal effusion were also noted. A caudal vena cava thrombus with secondary hepatic congestion and ascites was prioritized with neoplasia being a less likely differential diagnosis. Two liters of serosanguinous fluid were drained from the abdominal cavity, while the dog was under general anesthesia in preparation for the thrombectomy the following day. The dog was prepared for surgery, and a combined median sternotomy and a midline laparotomy was performed to approach the vena caval lesion. Upon inspection of the vena cava, a hard intraluminal mass invading the vessel wall was palpated. Intraoperative fine-needle aspiration of the lesion was performed with a 25-g needle. Cytologic examination revealed clusters of cells with poorly distinct cytoplasmic margins and round to rarely ovoid nuclei with finely coarsely stippled chromatin. Some clusters of cells had a minimal amount of pink extracellular matrix. These findings were consistent with a neoplastic process, and a diagnosis of possibly neuroendocrine neoplasia was favored by the pathologist on duty.

**Figure 3:**
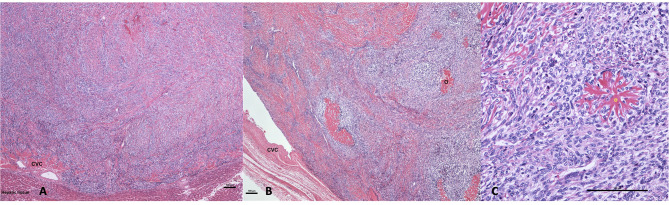
**(A)** Hematoxylin and eosin 40×: a neoplastic spindle cell proliferation filling the lumen of the caudal vena cava (CVC) where the neoplastic cell permeate the wall of the vein abutting hepatic tissue. **(B)** Hematoxylin and eosin 40×: luminal osteosarcoma filling the lumen and partially attached to the wall of the caudal vena cava (CVC). The neoplasm comprises multifocal deposition of osteoid (O). **(C)** Hematoxylin and eosin 200×: the neoplasm is composed of mitotically active, plum spindle to stellate cells that are multifocally centered on branched eosinophilic osteoid material (O) (bar is equal to 100 μm in each picture).

Due to the irresectability of the mass, poor clinical condition of the dog, and the suspected neoplastic process, the owners elected to euthanize the dog under general anesthesia and consented to perform a necropsy. The gross necropsy findings revealed a slightly enlarged and diffusely congested liver with rounded edges and a 5 cm × 2 cm-firm, tan, proliferative mass expanding and completely occluding the vena cava and a branch of the left hepatic vein. On microscopic examination, the caudal vena cava mass was composed of spindle-to-stellate neoplastic cells organized in streams and whorls embedded within mildly fibrous stroma that contained multiple branched fibrillar eosinophilic material consistent with osteoid (Figure 3). Immunohistochemistry for actin confirmed that the mass was intravascular within the liver vasculature. In the absence of a primary liver mass or other skeletal neoplasia, a diagnosis of primary EOSA of the caudal vena cava was established.

## Discussion

Intravascular neoplasia is rarely reported in the human literature, with leiomyosarcoma being the most common tumor arising from the inferior vena cava (15, 16). Osteosarcoma is another intravascular tumor that has been occasionally reported, arising from the pulmonary artery in humans (14). To the authors' knowledge, a primary intravascular neoplasm has yet to be described originating from the caudal vena cava in dogs. However, a case of an EOSA arising from the liver and invading the caudal vena cava has been previously reported (17). The current case differs from the reported hepatic-caval EOSA because no intraparenchymal portion of the mass was noted either on CT or on necropsy, making this mass exclusively a primary intravascular EOSA.

A definitive diagnosis of an intravascular tumor is often delayed in human medicine because of the difficulty of differentiating between the presence of mass from a bland thrombus and the rarity of these tumors making them unlikely to be considered as a differential diagnosis (15). The same situation happened in this case as the intravascular tumor was initially considered to be a bland thrombus caused by the hypercoagulable state related to the protein-losing enteropathy, and primary neoplasia was only suspected at the time of surgery.

Commonly, a combination of different imaging techniques, such as ultrasound, CT, and magnetic resonance imaging, is used to obtain a definitive diagnosis of thrombus vs. intravascular neoplasia (15). Bland thrombi, tumor thrombi, and primary intravascular tumors can all show evidence of vascularization on color Doppler because of the recanalization of the thrombus or the presence of vessels within the mass. Blood flow on color Doppler was not noted in this case. The lack of blood flow on color Doppler has also been described in a case of an inferior vena cava leiomyosarcoma in a human patient (18). Possible explanation for this finding is that the portion of the intravascular mass imaged was a bland thrombus created by the flow turbulence precipitated by the intravascular neoplasm (19). Furthermore, the tumor had poor vascularization that could not be identified with this imaging technique or the Doppler test was performed on a necrotic region of the mass.

When the CT angiogram was performed, the mass was minimal-to-no contrast enhancing compared to the pre-contrast images. This is in accordance with what was noted in a case of chondroblastic osteosarcoma of the pulmonary artery (14), and this feature together with the lack of a primary lesion rules out the presence of a tumor thrombus. The poor vascularity of the mass in these two cases could explain the CT findings. On CT images, the focal marked dilation of the caudal vena cava and the presence of mineralization within the thrombus could have raised concerns for a primary intravascular neoplasia rather than a bland thrombus, even if cases of mineralized chronic bland thrombi, due to dystrophic mineralization, have been reported (20).

A potentially more accurate imaging test used to differentiate between a bland thrombus or intravascular tumor is Fluorine-18 fluorodeoxyglucose positron emission tomography (^18^F FDG PET-CT) (19). Because of the usually high metabolic activity of the tumor, the radioactive glucose analog used as a tracer is accumulated within the tumor and can be easily identified on imaging using ^18^F FDG PET-CT (18). However, due to ^18^F FDG PET-CT's high sensitivity but extremely low specificity, septic and inflammatory thrombi can have a similar imaging appearance to intravascular neoplasia (19).

Negative prognostic factors reported for human patients affected by inferior vena cava tumors are the presence of Budd–Chiari syndrome, suprahepatic location of the tumor, intraluminal tumor growth, and inferior vena cava occlusion (16). The current patient had all those negative prognostic indicators. Additionally, the mass was deemed unresectable intraoperatively, justifying the decision of euthanasia. On necropsy, the mass was confirmed to arise from the caudal vena cava wall, and no additional lesions were identified within the liver, musculoskeletal system, and other major vessels. Having a preoperative full-body CT scan or an ^18^F FDG PET-CT would have been a more accurate method of staging to confirm that the caval mass was the primary with no other neoplastic lesions as small osseous lesion could have been missed on necropsy.

In conclusion, this case report describes for the first time in veterinary literature the presence of an EOSA arising from the post-hepatic caudal vena cava in a dog. Even if rare, EOSA should be added to the list of potential differentials in animals with intravascular neoplasia. The difficulty in diagnosing these EOSAs stems from the rarity of these tumors. Because of their asymptomatic nature, they remain silent for most of the disease course and are usually found due to their mass occlusive or space-occupying effect, as in human cases (21). Surgical resection of the mass when possible, or a representative biopsy, is needed to achieve a definitive diagnosis. Veterinarians, particularly diagnosticians, should be aware of the possibility of intravascular ESOAs, which will necessitate surgical resection as the primary choice of treatment especially when vascular neoplasia is suspected.

## Data availability statement

The datasets presented in this article are not readily available because, this is a case report, no data set was produced. Bloodwork and imaging of the case can be shared upon reasonable request. Requests to access the datasets should be directed at: giovanni.tremolada@colostate.edu.

## Ethics statement

Ethical approval was not required for the studies involving animals in accordance with the local legislation and institutional requirements because the animal was treated according to standard of care and no experimental procedures were performed. Written informed consent was obtained from the owners for the participation of their animals in this study. Written informed consent was obtained from the participant/patient(s) for the publication of this case report.

## Author contributions

GT prepared the first draft. GT, AM, LG, and TA managed the patient or interpreted the imaging tests or performed the necropsy/interpreted the histopathology and contributed to conception of the case report. All authors contributed to manuscript revision, read, and approved the submitted version.
